# A modified experimental model of malignant pleural disease induced by lung Lewis carcinoma (LLC) cells

**DOI:** 10.1186/s12967-015-0662-2

**Published:** 2015-09-15

**Authors:** Milena Marques Pagliarelli Acencio, Juliana Puka, Evaldo Marchi, Leila Antonangelo, Ricardo Mingarini Terra, Francisco Suso Vargas, Vera Luiza Capelozzi, Lisete Ribeiro Teixeira

**Affiliations:** Pleura Laboratory, Pulmonary Division, Heart Institute (InCor), University of Sao Paulo Medical School, Rua Dr. Eneas de Carvalho Aguiar, 44, Cerqueira César, São Paulo, Zip code: 05403-000 Brazil; Medical College of Jundiai, São Paulo, Brazil; Clinical Laboratory and LIM 03, Department of Pathology, Hospital das Clínicas, University of Sao Paulo Medical School, São Paulo, Brazil; Division of Thoracic Surgery, Heart Institute (InCor), University of São Paulo Medical School, São Paulo, Brazil

**Keywords:** Lewis lung carcinoma, Malignant pleural effusion, Lung cancer

## Abstract

**Background:**

Malignant pleural effusion resulting mainly from pleural metastases of lung adenocarcinoma has clinical relevance, being a sign of poor prognosis and low life expectancy. Experimental models can mimic the human condition, contributing to advances in current understanding of the mechanisms patients’ pleural fluid accumulation and possible therapeutic strategies. The objective of this study is to evaluate the role of different concentrations of Lewis lung carcinoma cells (LLC cells) at the time of induction of experimental MPE and the main effects on survival of animals.

**Methods:**

C57BL/6 mice received intrapleural injection of 0.1, 0.5 or 1.5 × 10^5^ LLC cells and survival curve, biochemical and pathological analyses of pleural fluid and tissue were analyzed.

**Results:**

Evaluation of weight loss, mobility and survival showed that animals that received 0.5 × 10^5^ cells maintained more stable condition up to day 14 and a gain of 6 days survival over mice that received the highest concentration.

**Conclusion:**

This study may allow a better understanding the mechanisms involved in the development of malignant pleural effusion and it may be promising in evaluating therapy to avoid recurrence, as the best time to indicate pleurodesis or target therapies.

## Background

Malignant pleural effusion (MPE) resulting from pleural metastasis of lung adenocarcinoma is a common clinical problem with severe implications, since it is a debilitating condition associated with high morbidity, poor prognosis and low life expectancy (3–15 months) [1–6]. Approximately 15 % of lung cancer patients present pleural effusion at the time of diagnosis and half of them develop pleural effusion at disease advanced stages [1–5].

Current therapeutic options for MPE are limited to treatment of the primary tumor and pleural cavity drainage with or without pleurodesis, practices that can cause pain and discomfort, carry risks of adverse effects, and do not benefit a substantial portion of patients [7–14].

For a long time, the pathogenesis of MPE has been poorly understood, but substantial progress has been made over the past few years facilitated by the use of animal models [15–19]. These models can mimic the human condition, contributing to advances in current understanding of the mechanisms patients’ pleural fluid accumulation and possible therapeutic strategies [19–27].

The objective of this study is to evaluate the role of different concentrations of Lewis lung carcinoma cells (LLC cells) at the time of induction of experimental MPE and the main effects on survival of animals.

The increase of survival time and delaying systemic effects, a better and more detailed understanding of the mechanisms involved in the development of malignant pleural effusion can be gained. This would facilitate future studies making a better assessment of therapeutic response possible.

## Methods

### Cell culture

The Lewis lung carcinoma (LLC) cells were purchased from the American Type Culture Collection (Manassas, VA, USA) and were cultured at 37 °C in 5 % CO_2_^−^ 95 % air using Dulbecco’s modiWed Eagle’s medium (DMEM) with 10 % fetal bovine serum.

### Animal model

One hundred and thirty male (6–8 weeks old) C57BL/6 mice (obtained from Laboratory Animal Center of Faculty of Medicine of University of São Paulo) were acclimatized for 1 week. All animal care and experimental procedures were approved by the University Ethics Committee (CEUA/CAPPesq).

Animals were anesthetized using 35 mg/kg of ketamine hydrochloride (Cristalia, Brazil) and 5 mg/kg of xylazine hydrochloride (Bayer, Brazil) prior to all procedures. The right chest was cleansed with an alcohol solution (Rioquimica, Sao Paulo, Brazil). The intrapleural injection was performed using a 23-gauge needle attached to a 1-mL syringe containing the solution of cells which was introduced into the chest cavity at 1 cm lateral to the right parasternal line. The plunger of the syringe was removed and the needle was slowly advanced until it reached the pleural space, where the sub-atmospheric intrapleural pressure allowed the fluid to enter the pleural cavity spontaneously. The mice were monitored after the procedure until they were completely recovered.

Three groups of 40 mice each received concentrations of LLC at 0.1, 0.5 or 1.5 × 10^5^ cells intrapleurally. These animals were subdivided into two groups; the first (30 animals per concentration of cells) were euthanized after 7, 14 or 21 days and the second group (10 animals per concentration of cells) were evaluated for survival expectancy. A control group of 10 animals received saline solution intrapleurally.

Mice were killed according to the study calendar; the abdominal wall was opened and the viscera were retracted to visualize the diaphragm. Pleural fluid (PF), when present, was gently aspirated and the volume was measured and placed in tubes for evaluation.

### Weight, mobility and survival analysis

After the procedure all animals were observed until complete recovery and they were evaluated for weight (g) and mobility by a subjective score of 0–3 (0 = normal and 3 = stillness). We monitored mortality daily for all groups to obtain the survival curve.

### Histological assays

After 7, 14 or 21 days the thorax was dissected and removed en bloc. A small amount of 10 % formaldehyde was injected through the trachea to keep the lungs expanded and the entire block plus kidney, liver and spleen were placed in 10 % formaldehyde. After at least 24 h in formaldehyde, the pleural cavities were opened and exposed through longitudinal chest incisions at the mid-clavicular lines. Tissues were fixed in 10 % neutrally buffered formalin for 24 h and 70 % ethanol for 3 days. In sequence, they were embedded in paraffin, and 5-μm thick slices were cut, mounted on slides and stained with hematoxylin and eosin (H&E).

### Biochemical assays

Lactic dehydrogenase (kinetic UV method) and total protein (Biuret method) were quantified in the pleural fluid using commercial kits (Wienner, Argentina) and analyzed in semi-automatic device.

### Cytology

Pleural fluid cells were counted in a Neubauer chamber. After centrifugation, cells cytospin were prepared and the slides were air dried and stained using Leishman staining to determine the cell differential.

### Statistics

The results are presented as mean and standard deviation. Comparisons among the groups were performed using ANOVA followed by the comparison multiple test. For the survival time, Kaplan–Meier curves were established for each group and the times were compared using a log-rank test. A value of *p* < 0.05 was considered significant. SigmaStat 3.1 (Systat, CA, USA) was used for the analyses.

## Results

After the intrapleural injection of different concentrations of LLC cells, all animals presented tumor implantation with development of malignant pleural effusion showing differences among them according to the time and stage of disease. Free-floating bilateral pleural effusions and pleural tumor foci were clearly visible through the diaphragm. Interestingly, in the majority of mice that received 0.1 × 10^5^ cells, minimal or absence of pleural fluid and few tumoral implants were observed until the 14th day.

Evaluation of body weight and mobility showed that mice in the group of 1.5 × 10^5^ cells had reduced weight and mobility after 14 days while in the other groups, these differences were noted only after 21 days (p < 0.05).

**Fig. 1:**
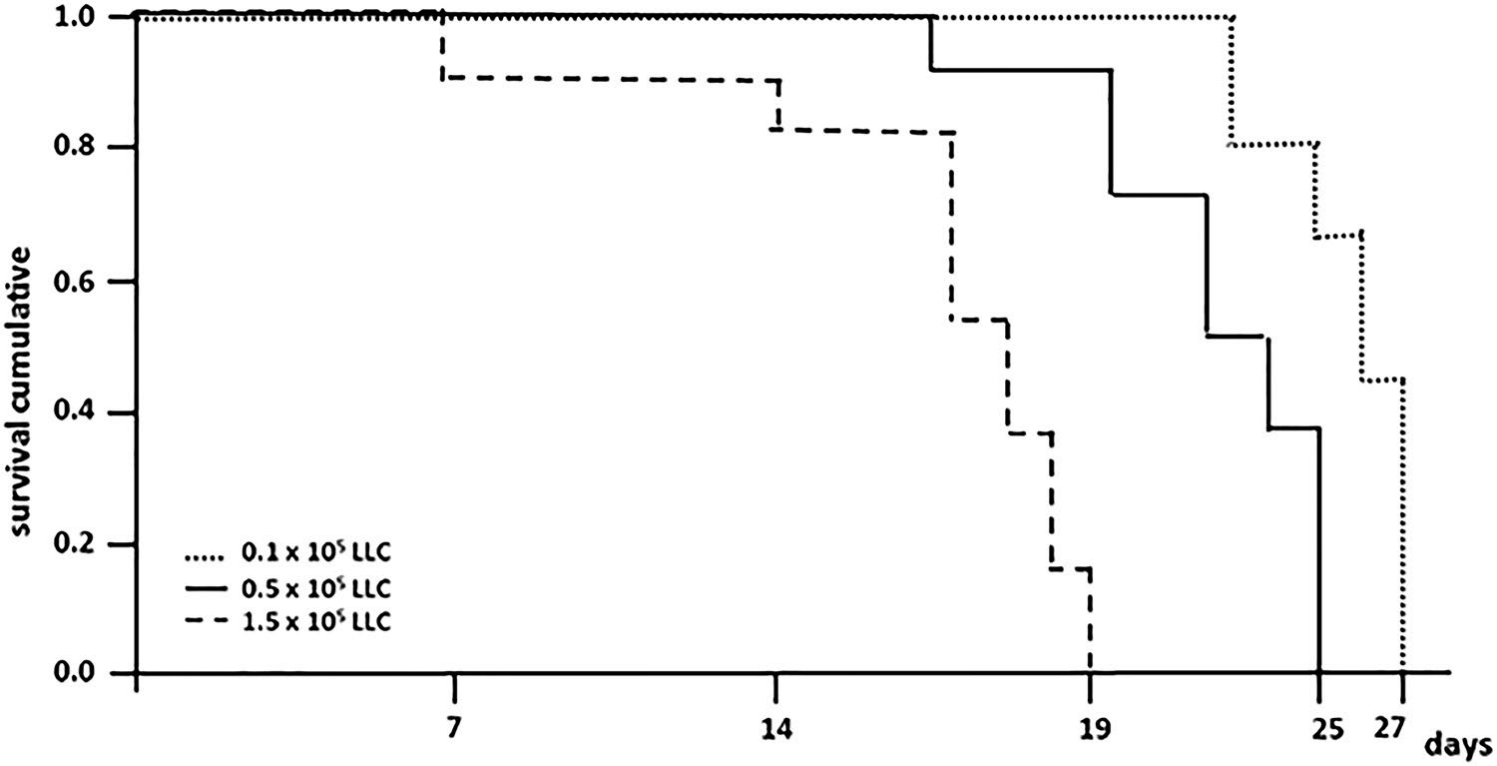
Mice survival time evaluation with intrapleural injection of 0.1, 0.5 or 1.5 × 10^5^ LLC cells

In the survival analysis, pleural carcinomatosis was lethal in all groups. In the group of highest concentration (1.5 × 10^5^ cells) the mortality was 100 % on the 19th day while the groups of 0.5 and 0.1 × 10^5^ cells showed maximum survival of 25 and 27 days, respectively (Fig. 1).

**Fig. 2:**
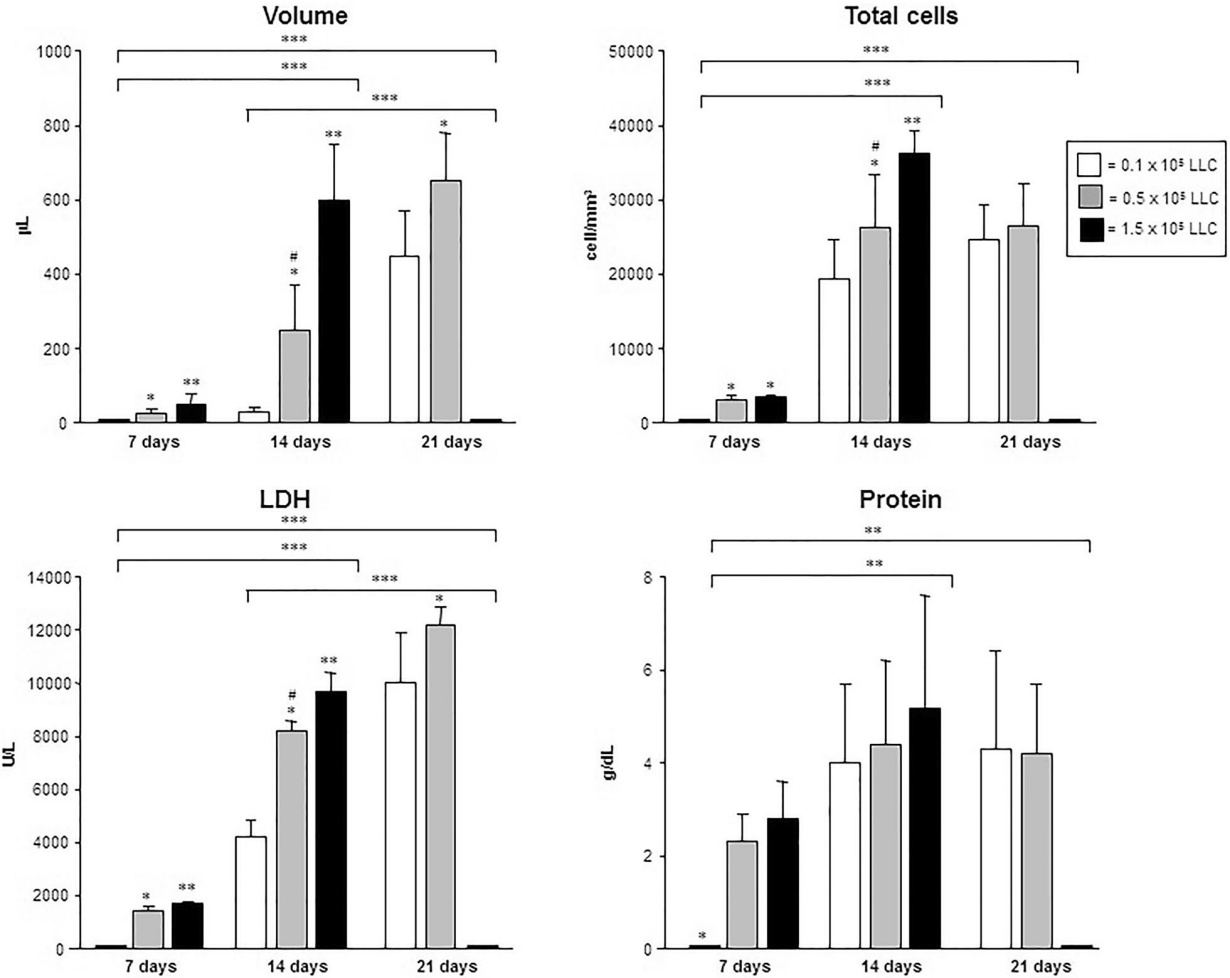
Volume, total cells count and biochemical parameters of malignant pleural effusion in mice injected with 0.1, 0.5 and 1.5 × 10^5^ LLC cells. *p < 0.05, **p < 0.001, ***p < 0.001 and ^#^p < 0.05 when compared the groups

Pleural fluid volume was measured at 7, 14 and 21 days. The volume was increasing significantly according to the time and LLC cells concentration (p < 0.05); group 0.1 × 10^5^ cells showed pleural effusion only after 14 days in few animals and for group 1.5 × 10^5^ cells, evaluation was only possible up to the 14th day (all animals died by day 19) (Fig. 2). In the control group (saline intrapleurally), we do not observe pleural fluid accumulation.

The macroscopic aspect of most pleural fluid was hemorrhagic (but did not coagulate). By harvesting mice at serial time points we observed that malignant pleural effusion was formed gradually and that their red blood cell content increased stepwise.

Total cells count was progressively increasing up to 14 day being more evident in highest concentrations (0.5 and 1.5 × 10^5^) (Fig. 2). MPE differential cells revealed a mixed inflammatory infiltrate interspersed with malignant cells. The inflammatory cell population consisted of mononuclear cells (60 %), lymphocytes (20 %) and neutrophils (10 %). Mesothelial cells accounted for less than 1 % of cells.

Pleural fluid LDH levels were higher proportional to the time (p < 0.001) and concentration (p < 0.05) of injected LLC cells. Protein levels were similar among groups excepted at 7 days in 0.1 × 10^5^ LLC cells group (Fig. 2).

**Fig. 3:**
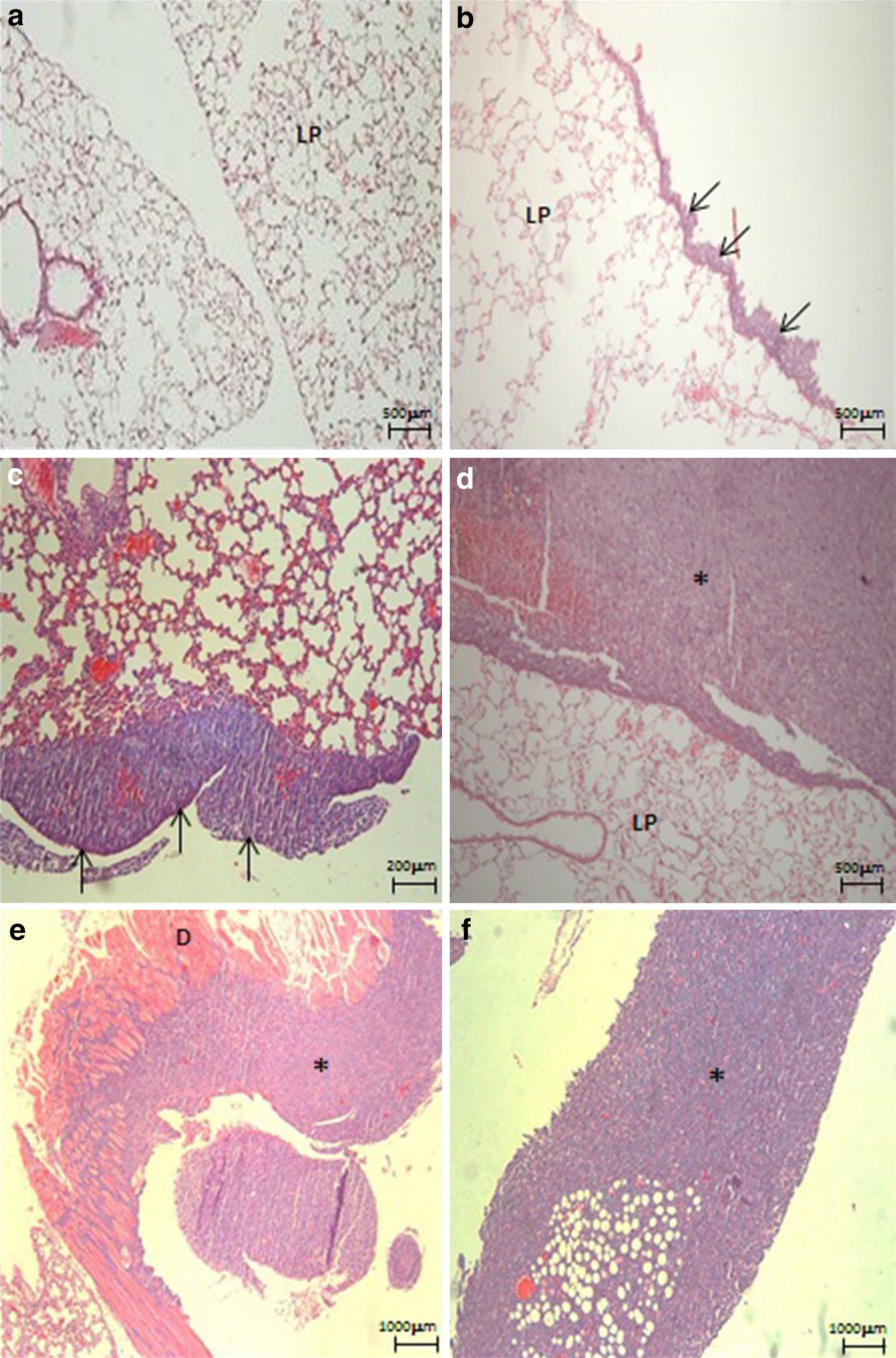
Histology of pleura, lung parenchyma and diaphragm of mice that received intrapleural injection of LLC cells. **a** Pleura and lung parenchyma after 7 days of 0.1 × 10^5^ cells; **b** tumor implantation in visceral pleura after 7 days of 0.5 × 10^5^ cells (*arrows*); **c** tumor implants in visceral pleura after 14 days of 0.5 × 10^5^ cells (*arrows*); **d** tumor implants in visceral pleura after 14 days of 1.5 × 10^5^ cells (*asterisk*); **e** massive tumor invading diaphragm after 14 days of 1.5 × 10^5^ cells (*asterisk*); **f** tumor mass observed after 21 days of LLC cells (*asterisk*). Hematoxylin & eosin staining. *LP* lung parenchyma, *asterisk* tumor, *arrows* tumor implants

Tumor implants in the pleura were evident earlier in groups that received a larger concentration of LLC cells. The presence of loose tumors in the pleural space was also noted in all study groups at 7 days except in the group with the lowest concentration of LLC cells. Larger pleural tumors formed bridges between the lung parenchyma and the thoracic cage and infiltrated neighboring anatomic structures, including the chest wall, mediastinum, and diaphragm (Fig. 3). In group 1.5 × 10^5^ LLC cells numerous tumor implants were observed in the visceral and parietal pleura after 7 days of exposure. In group 0.5 × 10^5^ cells LLC we noted few dispersed implants at day 7 with more pronounced implantation after 14 days. In animals receiving lower cells concentration, tumor implants were only observed after 14 days (Fig. 3).

Neoplastic infiltration of lung parenchyma was observed only in few animals with no correlation with the dose or exposure time. However, lung parenchymal inflammation was unremarkable in all groups over all check points; there was no inflammatory focus at concentration 0.1 × 10^5^ cells on day 7.

**Fig. 4:**
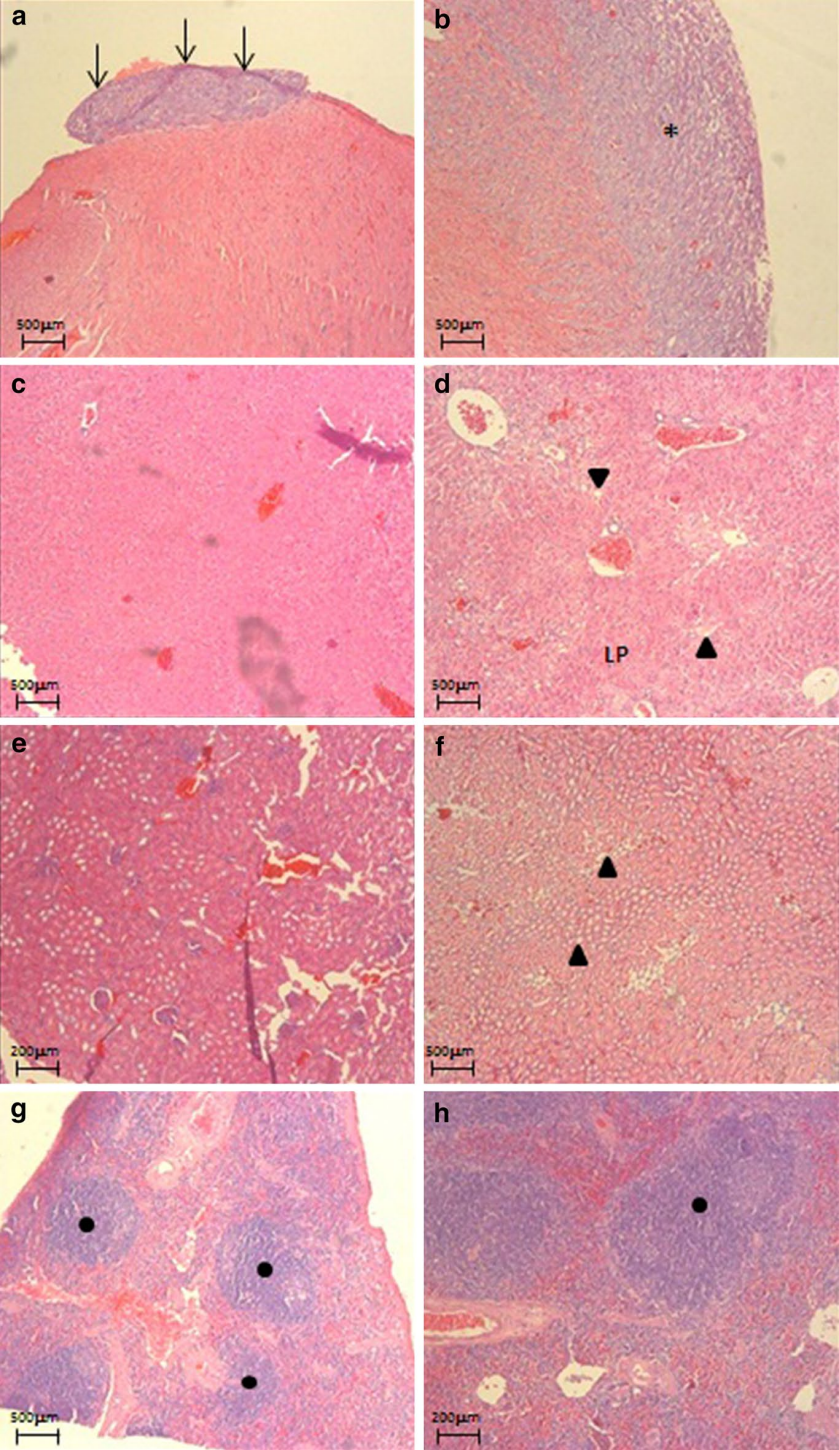
Histology of organs of mice that received intrapleural injection of LLC cells. **a** Tumor implants in pericardium after 7 days of 1.5 × 10^5^ cells (*arrows*); **b** neoplastic cells infiltrating the myocardium (*asterisk*) after 14 days of 1.5 × 10^5^ cells; **c** normal liver after 7 days of 0.5 × 10^5^ cells; **d** liver with steatosis (*filled triangle*) after 14 days of LLC cells; **e**, **f** kidney with tubular steatosis (*filled triangle*) and without neoplastic cells after 7 or 14 days. **g**, **h** Reactive spleen (*filled circle*) without neoplastic cells after 7 and 14 days. Hematoxylin & eosin staining

Histological evaluation of pericardium and heart muscle showed tumor implants in the first 7 days after injection of 1.5 × 10^5^ LLC cells with increased progression over time. In the other concentrations few tumor implants were observed during the study (Fig. 4).

In the liver, micro foci of steatosis were observed after 14 days at concentrations 1.5 and 0.5 × 10^5^ LLC cells. No relevant histological changes were observed in the renal evaluation. Hyperplasia of the white pulp of the spleen was observed at all evaluation time points with greater evidence at concentrations of 1.5 and 0.5 × 10^5^ LLC cells (Fig. 4).

## Discussion

This study evaluated the survival curve, laboratorial and histological aspects in an experimental murine model of MPE using different concentrations of LLC cells. Evaluation of weight loss, mobility and survival at the higher concentrations (0.5 and 1.5 × 10^5^ cells) showed that animals that received 0.5 × 10^5^ cells maintained more stable condition up to day 14. The survival curve shows a gain of 6 days survival over mice that received the highest concentration. We found MPE from day 7 and several tumor foci in both the visceral and parietal pleura in the higher concentration groups. However, minimal or no volume of pleural fluid and few implants were observed in the smaller LLC concentration group at day 14.

Lung cancer is the most common cause of worldwide cancer-related mortality, leading to over a million deaths each year and adenocarcinoma is its most common histological type [1–5]. In this scenario, MPE resulting mainly from pleural metastases of lung adenocarcinoma has clinical relevance [4, 14]. Its emergence is a sign of poor prognosis and low life expectancy, as it indicates that the tumor is incurable by surgery [4]. Dyspnea occurs in more than 50 % of patients, severely compromising their quality of life. In spite of the therapeutic advances since the platinum-based schemes, lung cancer treatment does not significantly impact the disease evolution of most patients [4–14].

New therapeutic strategies are needed to improve local control and the spread of cancer, but this requires greater insight into the pathogenesis of this disease.

Until some time ago, there were a few experimental models for the study of pleural neoplasms, most using animals with normal pleura to test palliative therapies such as pleurodesis [24–26]. In the absence of malignant disease assessment of the natural evolution of the cancer, neoplasia-host interactions, or possible immune response is obviously not possible [25, 26]. Therefore, some researchers have developed novel models of MPE with a more accurate mimicry of the human condition. These models have contributed to significant advances in the understanding of fluid exudation induced by cancer metastasis to the pleura [19]. They have yielded novel insights into the pathogenesis of adenocarcinoma as well as into the mechanisms of intrapleural malignant effusion accumulation, tumor dissemination and possible therapeutic targets to block pleural effusion [19].

In 2006, Stathopoulos et al. developed and characterized a novel mouse model of malignant pleural effusion by injecting 1.5 × 10^5^ Lewis lung carcinoma (LLC) cells directly into the pleural space of immunocompetent C57BL/6 mice [20].

LLC cells are derived from a spontaneously arising lung adenocarcinoma in C57BL/6 mice. These cells are characterized by short doubling times in vitro and in vivo and aggressive biological behavior [19–21]. The cells can be propagated in C57BL/6 mice, giving rise to lung adenocarcinoma and human-like MPEs producing exudates with high protein and LDH content, as well as high levels of VEGF and monocyte chemoattractant chemokines [19–21].

This LLC-C57BL/6 model is highly reproducible since it uses immunocompetent animals, closely resembles human MPEs and provides reproducible end-points. It can be used to study the influence of specific host and tumor factors on the pathogenesis of MPE and to evaluate new therapeutic strategies. In addition, all of the animals get MPE and intense tumor foci on visceral and parietal pleura in 14 days [20].

Nevertheless, the maximum survival of the animals in the Stathopoulos et al. model was 17 days, an unfavorable point, since survival is an important outcome measure and so few days may not be sufficient to assess outcome [20, 21].

Also, in these studies authors observed a correlation between the number of pleural tumor foci and the volume of pleural fluid. They discussed the importance of the host immune response, which is partly responsible for the malignant pleural fluid accumulation [20–23]. These issues are directly relevant in studies to investigate the pathogenesis and treatment of MPEs. The pleural fluid accumulation remains the primary end-point in this model; however, determinations relevant to these bio-processes may serve as additional end-points in animal models of MPE.

The cellular and biochemical profile of pleural effusion resulting from induced lung adenocarcinoma mimics human MPE, which is high in protein and LDH levels and nucleated cells with neoplastic cells permeating a rich inflammatory infiltrates. Implantation and growth of pleural tumors triggers a host inflammatory response characterized by a mixed inflammatory cell influx into the pleural fluid, angiogenesis, and vascular hyperpermeability, all fundamental steps in MPE pathogenesis [19–21].

We found tumors implants in the visceral and parietal pleura in all groups studied and they were proportional to the concentration and time from instillation of LLC cells. Metastasis to the pericardium was more evident at the highest concentration. Inflammatory lung parenchyma cells and histological changes in the liver, kidneys and spleen were observed in all groups with the highest scores in groups 0.5 and 1.5 × 10^5^ cells.

Furthermore, induction of MPE in animals leads to cachexia, which can serve as a surrogate marker of tumor progression. In our study mice that received 0.5 × 10^5^ LLC cells maintained more stable condition through day 14 compared to mice that received the highest concentration, resulting in a longer survival.

One limitation of our study was related to the assessment of animals at 21 days due to the fact that most died before analysis could be done.

## Conclusions

Our results show that using a dose of 0.5 × 10^5^ cells LLC is possible not only to induce the disease as well as to better evaluate survival time, without prejudice to the model proposed initially by Stathophoulous and colleagues [20]. With this cell concentration we obtained a model that allowed for monitoring longer survival, the study of pleuropulmonary changes and to assess local and distant metastases, opening a fantastic range of study opportunities.

This study may allow a better understanding the mechanisms involved in the development of malignant pleural effusion. In addition, it may be promising in evaluating therapy responses, the best time to indicate pleurodesis, as well as, to consider the response to immunomodulatory therapies, costs and benefits of targeted therapies.
